# Stem Cell Models and Gene Targeting for Human Motor Neuron Diseases

**DOI:** 10.3390/ph14060565

**Published:** 2021-06-12

**Authors:** Yashashree Karpe, Zhenyu Chen, Xue-Jun Li

**Affiliations:** 1 Department of Biomedical Sciences, University of Illinois College of Medicine, Rockford, IL 61107, USA; ykarpe2@uic.edu (Y.K.); zychen12@uic.edu (Z.C.); 2 Department of Bioengineering, University of Illinois at Chicago, Chicago, IL 60607, USA

**Keywords:** human pluripotent stem cells, motor neuron diseases, gene editing, neurodegeneration, axonopathy, amyotrophic lateral sclerosis, spinal muscular atrophy, gene therapy, antisense oligonucleotides, viral vectors

## Abstract

Motor neurons are large projection neurons classified into upper and lower motor neurons responsible for controlling the movement of muscles. Degeneration of motor neurons results in progressive muscle weakness, which underlies several debilitating neurological disorders including amyotrophic lateral sclerosis (ALS), hereditary spastic paraplegias (HSP), and spinal muscular atrophy (SMA). With the development of induced pluripotent stem cell (iPSC) technology, human iPSCs can be derived from patients and further differentiated into motor neurons. Motor neuron disease models can also be generated by genetically modifying human pluripotent stem cells. The efficiency of gene targeting in human cells had been very low, but is greatly improved with recent gene editing technologies such as zinc-finger nucleases (ZFN), transcription activator-like effector nucleases (TALEN), and CRISPR-Cas9. The combination of human stem cell-based models and gene editing tools provides unique paradigms to dissect pathogenic mechanisms and to explore therapeutics for these devastating diseases. Owing to the critical role of several genes in the etiology of motor neuron diseases, targeted gene therapies have been developed, including antisense oligonucleotides, viral-based gene delivery, and in situ gene editing. This review summarizes recent advancements in these areas and discusses future challenges toward the development of transformative medicines for motor neuron diseases.

## 1. Introduction

Stem cells are essential for living organisms and possess two unique characteristics: self-renewal and differentiation potential [1]. Depending on the potency, stem cells are classified into totipotent, pluripotent, multipotent, oligopotent, and unipotent stem cells. Among these different types, pluripotent stem cells (PSC) have been used extensively for a variety of applications. PSCs can form all cells from the endoderm, mesoderm, and ectoderm but cannot form the extra-embryonic cells like the placenta [2,3]. In the developmental stages of a zygote, the formation of a blastocyst occurs with pluripotent embryonic stem cells (ESCs) present in the inner cell mass. The successful establishment of human ESC lines from blastocysts was first reported by James Thomson and the group in 1998 [4]. They showed that these cells have normal karyotypes and possess the property of self-renewal and can differentiate into three germ layers.

The other type of human PSC is induced pluripotent stem cells (iPSCs), which are reprogrammed from somatic cells. In 2007, Shinya Yamanaka and his group successfully reprogrammed human fibroblast cells into iPSCs, by introducing four pluripotent factors including Octamer-binding transcription factor (Oct-3/4), Sex determining region Y-box 2 (Sox2), c-Myc, and Kruppel-like factor 4 (Klf4) [5]. The reprogrammed cells exhibited characteristics morphology similar to ESC. More importantly, they express pluripotent proteins and can form three germ layers both in vitro and in vivo, confirming their pluripotency. By introducing Oct4, Sox2, Nanog, and Lin28, James Thomson and his group also generated stable iPSCs from human somatic cells [6]. Now scientists can generate integration-free iPSCs from multiple cell types including fibroblast cells and blood cells [7,8,9,10]. These iPSCs, which maintain the genetic mutations in somatic cells, have the potential to generate the specific type of cells affected in patients, providing unlimited patient-specific cells for studying a variety of diseases [11,12,13,14].

Neurodegenerative diseases are characterized by the degeneration of specific neuronal subtype(s). For example, spinal (lower) motor neurons are affected in Spinal Muscular Atrophy (SMA), cortical (upper) motor neurons are affected in Hereditary Spastic Paraplegias (HSPs), and both spinal and cortical motor neurons are affected in Amyotrophic Lateral Sclerosis (ALS). To model these diseases, patient-derived iPSCs or genetically modified human ESCs are required to be differentiated into specific neuronal subtype affected in the disease. The differentiation of neuronal subtypes from PSCs is a multistep process that begins with the induction of neuroectoderm, then patterning the regional progenitors, and finally maturation of the post-mitotic neurons. By applying neural signaling pattern to mimic the development of these neurons during the embryonic stage, different neuronal subtypes have been generated from both hESCs and iPSCs [15,16] As an example, both caudalizing signal (Retinoic acid, RA) and ventralizing signal (sonic hedgehog, SHH; or SHH pathway activator, Purmorphamine) are required for specifying spinal motor neurons. Without caudalizing morphogens, PSCs undergo default pathway and differentiate into telencephalic glutamatergic neurons (i.e., cortical projections neurons) [17]. The detailed protocol for generating motor neurons, as well as other neuronal subtypes is out of the scope of this review and has been summarized previously [15,18,19,20,21].

Considering the difficulties to obtain human neural cells, human PSCs (hPSC) provide an unlimited source of human neuronal subtypes that can be used to study neural development and neurodegenerative diseases [14,22]. Patient-relevant motor neurons have been shown to recapitulate the human disease (both genetic and sporadic) phenotypes and are also used for testing therapeutic agents. One major challenge in the hPSC field is the low efficiency of gene editing which contributes to the difficulty in studying the role of a particular gene or protein. With the development of gene editing technologies like Zinc-Finger Nucleases (ZFN), Transcription Activator-Like Effector Nucleases (TALEN), and Clustered Regularly Interspersed Short Palindromic Repeats (CRISPR)-Cas9, the efficiency to edit human PSCs has increased dramatically.

The objective of this review is to discuss the role of stem cells and the different gene editing strategies in studying human neurological diseases with the focus on ALS and SMA. This review will introduce the recent advancements in gene editing, summarize the studies carried out using the PSC technology, and will discuss the applications of gene editing in stem cell models of motor neuron diseases. In addition, this review also discusses the recent developments in gene therapy including in vivo gene editing for treating these motor neuron diseases.

## 2. Traditional Gene Targeting Methods

Since the DNA double helix was discovered, there was a need to search for technologies that could precisely manipulate it. The highly specific gene engineering tools provide the power to characterize gene functions and correct gene mutations for the eradication of genetic diseases. It also helps to establish transgenic animal models and cell lines for studying complex disease mechanisms. The gene targeting methods introduce double-stranded breaks (DSBs) in the DNA and these are repaired by two different repair processes namely, Non-Homologous End Joining (NHEJ) and Homology Recombination (HR). The NHEJ is a quick, error-prone process that frequently induces insertions and deletions (indels) at the site of the break. This process utilizes Ku proteins to attach at the break site and employ different proteins including nuclease, polymerase, and DNA ligase to join the two ends [23]. On the other hand, HR is a relatively error-free and highly sophisticated process. At the DSB, the resection of the DNA strand is coordinated by different proteins and produces 3′-OH single-stranded DNA (ssDNA). This ssDNA then does strand invasion into the sister chromatid and uses it as a template for DNA synthesis. Following strand invasion, the repair can occur either through Holliday junction formation or synthesis-dependent strand annealing, leading to precise and error-free DNA repair [24]. Although it is specific, the frequency of recombination is very low (~1 in 10^9^ cells) [25]. With the development of artificial recombinase systems including ZFNs, TALENS, and CRISPR-Cas9, the efficiency of targeting human genes is significantly increased thus, greatly facilitating the study of human diseases.

### 2.1. Zinc Finger Nuclease (ZFN)

It has been shown that the restriction-modification enzymes are capable of performing the dual-action: recognizing the target DNA sequence and cutting at a specific site. However, there was a need for Type II restriction-modification enzymes that could recognize longer stretches of DNA. During the study of transcription initiation in Xenopus laevis, it was found that transcription factor IIIA was essential for correct initiation and was shown to interact with 50 nucleotides long internal control region [26]. When this factor was purified from oocytes, it revealed that this protein had nine repetitive zinc-binding domains called the Zinc Finger (ZF) motifs. Each motif is made of about 30 amino acids with pairs of cysteines and histidines (Cys_2_His_2_) that are stabilized after binding the zinc (Zn) ion [26]. Each of the ZF identifies 3-4 base pairs and inserts alpha-helix in the major groove of the DNA. When each of these fingers is placed in tandem, they can recognize a stretch of DNA with high specificity. These ZF domains can be linked to type II restriction enzyme *Fok I* (belonging to Flavobacterium okeanokoites) nuclease and therefore called Zinc Finger Nuclease.

**Figure 1:**
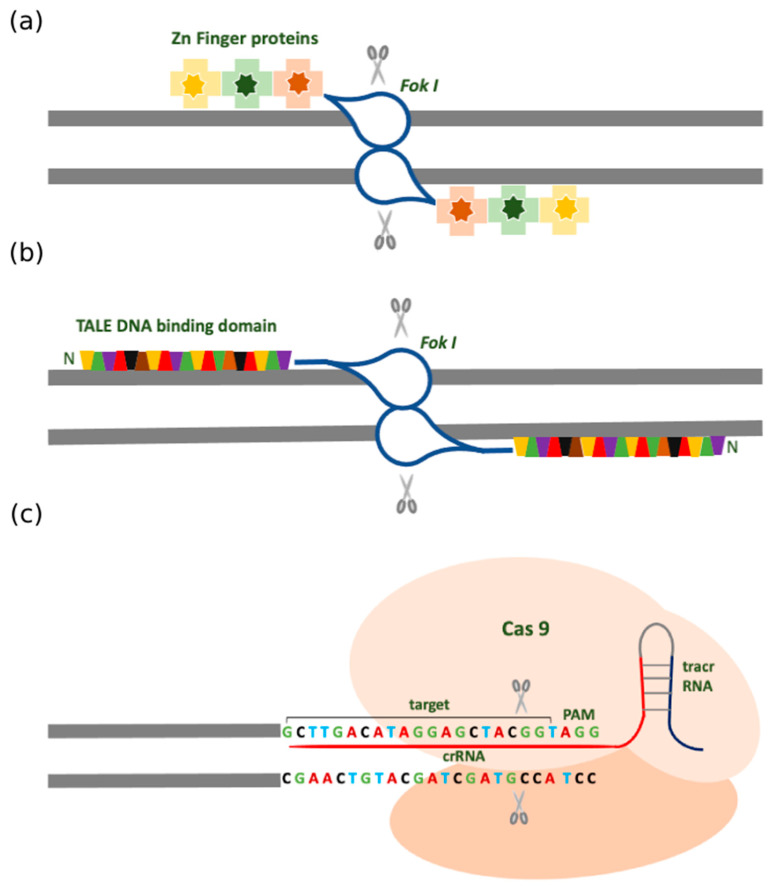
Schematic representation of the gene editing tools. (**a**) zinc-finger nucleases (ZFNs) have Zn Finger domain that recognizes and binds the DNA strand and is fused with the *Fok* I nuclease. The *Fok* I nuclease requires dimerization in the opposite orientation to nick the DNA. (**b**) TALENs comprise the TALE DNA binding domain fused with the nuclease. The amino acids at certain positions called repeated variable di-residues (RVDs) recognize one of three nucleotides in the DNA strand to nick the DNA. (**c**) CRISPR Cas9 system with the sgRNA that recognizes specific target sites. The crRNA binds and destabilizes the DNA double helix, while the tracrRNA recruits the Cas9 protein which recognizes the PAM site and nicks the target DNA sequence.

Preliminary studies pointed out that a hybrid endonuclease could be constructed comprising of different DNA binding proteins attached to the cleavage domain of *Fok I* leading to the discovery of chimeric restriction endonuclease ZFN [27]. To nick the target sequence, the ZFNs are designed as pairs that identify the flanking sequences, and subsequently, the dimerized *Fok I* introduces a DSB [27,28]. This dimerization requirement provides an extra level of specificity and defines the cut to be specifically at the center of the dimerized ZFN pair [29]. Earlier, three Zn finger DNA binding domains were used and gradually the number of Zn fingers increased up to six, recognizing longer stretches of DNA strands (Figure 1a) [30,31]. The genome of the cell repairs the nick by either error-prone NHEJ through shifting the open reading frame causing premature truncations and developing a knockout, or by efficient HR with a template comprising the homology arms. The double-stranded DNA vector or the single-stranded oligonucleotide can be used as a template for the HR-mediated repair process. For editing the human PSCs, the homology arms of 20 nucleotides are sufficient [32].

Despite these advancements, there are few drawbacks associated with ZFN. The ZFN design and engineering are technically challenging. It has low strand cutting efficiency and toxicity due to cleavage at off-target sites [33,34,35]. This is true especially in mammalian cells like iPSC and hESCs. Although there are no apparent karyotypic alterations in these cells [36,37,38], the ZFN-mediated gene editing may cause minor alterations, like indels, which may accumulate and become deleterious at a later stage.

Additionally, the ZFNs can be used every few hundred base pairs for binding limiting the target site selection. Despite these shortcomings, a remarkable application of this technology was for inactivating the Human Immunodeficiency Virus (HIV) coreceptors, C-C chemokine receptor 5 (CCR5), and C-X-C chemokine receptor 4 (CXCR4) in the CD4 T cells. Without the functional co-receptors, the CD4 T cells would become resistant to HIV and this could be regarded as the cure for the lethal Acquired Immune Deficiency Syndrome (AIDS) [39]. A recent study showed the HIV remission was possible with a single allogeneic hematopoietic stem cell transplantation with homozygous CCR532 donor cells [40].

### 2.2. Transcription Activator-Like Effector Nucleases (TALENs)

Based on the studies of the plant pathogen derived from the Xanthomonas genus, it was found that these bacteria secrete transcription activation-like effectors (TALE) proteins that can bind the DNA and regulate gene expression. TALE proteins are comprised of a DNA binding domain, nuclear localization sequence, and a gene transcription activating domain [41]. The DNA binding domain is made of monomers which are tandem repeats with 34 amino acids. The amino acids at positions 12 and 13 are highly variable and designated as repeated variable di-residue (RVD). These RVDs recognize the specific nucleotides in the target sequence [42,43]. At the 5′ end of the sequence bound by TALE proteins, the target sequence always has a thymine nucleotide affecting the binding efficiency [44]. The final tandem repeat that binds a nucleotide at the 3′ end of the target recognition site comprises only 20 amino acids and is called half repeat. After recognizing the structure of the DNA binding domain there were attempts to construct chimeric TALE nuclease. For this, a plasmid vector that was earlier used for ZFN was considered [45]. The vector consists of nuclear localization signal, half-repeat, N- terminal domain, *Fok I* domain, and an artificial DNA- binding domain is inserted. Since TALENs function as pairs, the target sites are selected on the opposite strands and are separated by 12-25 bp spacer. Once it reaches the nucleus and binds the target, the *Fok I* domains dimerize and introduce a double stranded break in the spacer (Figure 1b).

Both the DNA recognizing domain and the nuclease domain can be custom engineered to edit any sequence in the DNA. However, when compared to ZFN, this endonuclease is easier to design. The TAL repeats have been constructed de novo using the RVDs and seen to have a high affinity for the target sites [46]. TALEN-mediated gene engineering has been developed to construct reporter cell lines for lineage tracing [47] as well as to introduce point mutations [48] and study their effects. The target site selection is flexible with potential sites present every 100 base pairs [49]. The first-ever clinical TALEN application was for cell-based therapy to develop a Chimeric Antigen Receptor (CAR) on T cells by depleting both TCR and CD25. These engineered T cells can be used to treat Acute Lymphocytic Leukemia (ALL); however, it is an expensive therapy, and the patient should have enough healthy T cells [50]. The designing and construction of TALENs, on the other hand, is rapid and has strong gene targeting efficiency in human PSCs [51].

### 2.3. Clustered Regularly Interspersed Short Palindromic Repeats (CRISPR)

CRISPR was initially discovered in the adaptive immune system of bacteria against viruses and is the latest technology of gene editing [52]. The CRISPR-associated (Cas) proteins are nucleases with characteristic histidine and asparagine residues (HNH) responsible for introducing a DSB in the target DNA strand and RuvC-like nuclease responsible for the cleavage of the non-target strand [53]. There are three types of CRISPR (Type I-III) that have been well characterized till now, although there are six types and 29 subtypes reported in bacteria and archaea [54,55]. These three types are categorized based on the Cas proteins associated with them; type I has Cas3, type II has Cas9, and type III has Cas10 [55]. The type II CRISPR-Cas9 system from S. pyogenes is well-characterized and commonly used. It consists of SpCas9 nuclease, sgRNA, and a PAM site (NGG) downstream of the target site. The PAM site causes local destabilization of the DNA strand in order to form the DNA: RNA hybrid. The sequence of the PAM sites varies within different Cas proteins [56,57]. With the help of this system, NHEJ or HR pathways can introduce specific genomic modifications like insertions and deletions (indels).

The CRISPR-Cas9 system acts in an elegantly controlled and step-wise manner (Figure 1c). Firstly, the foreign DNA is cleaved into short fragments, which then integrates into the array of CRISPR RNA (crRNA) repeats. Secondly, this array is transcribed into pre-crRNA and then matures into crRNA. In type II CRISPR systems, the trans-activating CRISPR RNA (tracrRNA) hybridizes with the pre-crRNA, and is, therefore, necessary for target recognition and cleavage. The final step is mediated by Cas proteins that cleave the foreign genome and protect the host organism from infection. The crRNA and tracrRNA together constitute the single guide RNA (sgRNA or gRNA) and it renders the target specificity by Watson–Crick base pairing with the target site in the DNA strand [53]. By modifying the guide RNA sequence, new sites can be targeted and so the CRISPR-Cas9 system can be effectively used for high throughput applications.

Compared to ZFN and TALENs, the use of CRISPR-Cas9 has advantages [58]. Unlike ZFN and TALENs, it does not require the engineering of the proteins in the form of large DNA fragments for every new site. Rather, only the sequence of the 20-bp protospacer (i.e., the crRNA) needs to be altered by subcloning the altered nucleotide sequence into the gRNA plasmid. The nuclease Cas9 component need not be modified each time. In addition to this, this system exhibits the ability to multiplex. In other words, multiple gRNA can target various sites within a cell. Regarding the selection of the site for CRISPR-Cas9, a 23-bp sequence with the NGG PAM site at the end is required. Surprisingly, on average, this occurs once in every 8 bps. Another merit of the CRISPR-Cas9 system is that it is inexpensive and straightforward compared to the other gene editing systems. The CRISPR technology has proven to be an indispensable tool in research as it is highly efficient and scalable, due to its ability to edit multiple loci simultaneously.

The researchers have developed three approaches to facilitate genome editing using the CRISPR-Cas9. Firstly, the Cas9 and the sgRNA can be delivered directly into the cell rendering expeditious delivery, higher stability, and a low level of antigenicity [59]. The second approach involves a plasmid encoding sgRNA and Cas9 [60,61] which in vitro assembles both components, leading to their sustained expression and circumvents multiple transfections [62]. The introduction of this plasmid into cells is critical for successful transfection. The final approach is delivering the Cas9 mRNA and the sgRNA intracellularly. However, mRNA stability is of major concern since it may limit the duration of the gene modification [63]. Although, this technique seems promising for gene editing there are a few pitfalls that need to be improved with deriving efficient strategies. One of the major drawbacks is the S. pyogenes Cas9 protein size which is about 4.2 kb and is quite challenging for efficient in vivo delivery while using viral vectors. This is larger than TALEN and ZFN monomer. A solution to this is to use Cas9 proteins from other strains which are smaller in size.

### 2.4. Different Forms of CRISPR-Cas Systems

In order to upgrade the specificity of the Cas9-based gene editing, a Cas9 mutant was generated that works with paired offset sgRNAs which show complementarity to the strands opposite the target sequence [64]. This mutant form is named D10A mutant Cas9 nickase which has one of the catalytic domains inactivated and so it cleaves one of the double strands of the DNA helix. When paired with gRNA it leads to double nicking [65]. The paired nickase boosts the specificity but is not a dimerization-dependent system. They prefer just the co-localization of the two Cas9 nickases and each of them can introduce DNA nicks independently to incorporate mutations augmenting the chance of unwanted mutations. To control the frequency and the extent of undesirable off-target indels, dimeric RNA-guided *Fok I* nucleases (RFNs) have been developed. These are constructed by fusing the catalytically inactive dead Cas9 (dCas9) with *Fok I* nuclease. The RFNs require dimerization and two gRNAs for editing the genome. The N terminus of the dCas9 is fused with the *Fok I* nuclease domain unlike the ZFNs and TALENs which have the *Fok I* fused to the C terminus of the arrays. The gRNA/*Fok I*-dCas9 system functions efficiently with PAM sequences outside the target sites separated by an intervening 14–17 nucleotide spacer [65].

Taking into account the efficiency and simplicity of the CRISPR Cas system, there is an expansion of this system into a wide range of applications. Another type II CRISPR Cas system is CRISPR-Cpf1 (from Prevotella and Francisella 1) [66,67]. The PAM sequence that the Cpf1 crRNA recognizes is T-rich unlike the G-rich sequence for Cas9 and the Cpf1 efficiently cleaves the target site that precedes the T-rich PAM. Because Cpf1 recognizes different sequences compared with Cas9, this expands the capacity of gene targeting using CRISPR technology [68].

Traditional types of gene editing systems (Figure 1).

## 3. Application of Gene Editing in Stem Cell Models of Motor Neuron Diseases

**Figure 2:**
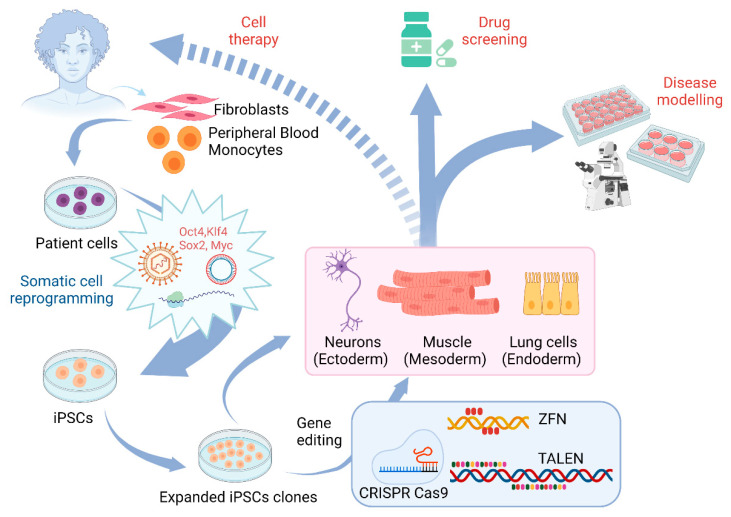
Generation of induced pluripotent stem cells (iPSCs) and their applications. The adult somatic cells are reprogrammed to form the iPSCs using the pluripotency factors. These iPSCs can be directly differentiated to cell types from the three germ layers and used for disease modeling, drug screening and cell therapy. Alternatively, the iPSCs can be edited by gene editing technologies for gene insertion, gene disruption, and gene correction. These gene edited iPSCs can then be used for further applications.

Pluripotent stem cells provide a unique source to generate specific neuronal subtypes to study neurodegenerative diseases that specifically affect these neurons including motor neuron diseases. Motor neurons are large projection neurons and include two types: upper motor neurons and lower motor neurons. Axons of upper motor neurons convey brain signals to lower motor neurons that innervate muscle cells to control the movement of the muscles. Motor neuron diseases are a group of neurological diseases that are characterized by the degeneration of motor neurons, leading to muscle weakness and atrophy. According to the National Institute of Neurological Disorders and Stroke, motor neuron diseases include Amyotrophic Lateral Sclerosis (ALS), Spinal Muscular Atrophy (SMA), Primary Lateral Sclerosis (PLS), Hereditary Spastic Paraplegia (HSP), Kennedy’s disease, and Progressive Bulbar Palsy (PBP). Though human PSC-based models of motor neuron diseases have been established, a great challenge is low efficiency of editing these human stem cells and their derivatives. With the development of gene editing technology, scientists can now knock-in specific mutations or correct the gene mutations to generate isogenic lines to study these diseases (Figure 2). Moreover, reporter lines can be generated using this technology to label motor neurons. The combination of gene editing techniques and stem cells offers unique opportunities for studying these diseases, as well as an opening to venture into the therapeutics area (Figure 2).

Generation and applications of induced pluripotent stem cells (Figure 2).

### 3.1. The Use of Gene Editing for ALS

ALS is a fatal neurodegenerative disease caused by the degeneration of both upper and lower motor neurons [69]. It is also named Lou Gehrig’s disease after an American baseball player who succumbed to ALS in 1939 [70]. Generally, the onset of this disease is in adulthood, and 3-5 years is the average time ALS patients survive after diagnosis. The neurodegenerative pathology for ALS includes excitotoxicity, hyperexcitability, neuroinflammation, mitochondrial dysfunction, axonal growth, functional defects, and dysregulated autophagy [70,71,72,73,74]. ALS patients include two categories namely familial ALS (FALS), and sporadic ALS (SALS). The majority of the cases are of SALS, whereas FALS comprises 10% of the total cases and is associated with mutations in multiple genes like *Superoxide Dismutase (SOD1), Fused in Sarcoma (FUS), TAR DNA-binding protein 43 (TDP43), Chromosome 9 open reading frame 72 (C9ORF72)* [70]. Currently, there is an FDA-approved drug used to combat the disease called riluzole. However, it only extends the life span for a short period [75]. There remains no cure for this disease at least partially due to the lack of human models to dissect the pathogenic mechanisms and screen for therapeutic drugs.

Since the successful establishment of iPSC from an ALS patient in 2008 [76], iPSC-based human models of ALS provide unique systems to study pathogenic mechanisms and to test compounds for therapeutic development [77,78,79,80]. In particular, motor neurons generated from iPSCs of sporadic ALS patients recapitulate disease-specific phenotypes, such as *TDP43* aggregations, offering tools to study various forms of ALS [77,81]. Considering that iPSCs from different individuals have a distinct genetic background, how to select appropriate controls is critical for the success of iPSC models. One major application of gene editing technology in stem cell models is to knock in or correct the disease mutation to generate isogenic lines. Since these lines have the same genetic background, isogenic pairs eliminate variations in genetic backgrounds and thus reduce the number of control lines that are needed for the discovery of pathological changes caused by gene mutations specifically. A group reported isolating the *SOD1* A272C mutation-bearing fibroblasts from an ALS patient and reprogramming them to become iPSCs [82]. They then employed CRISPR-Cas9 to rectify this mutation by providing a donor template that replaced this specific point mutation. After differentiating the iPSCs to motor neurons, significant differences in the gene expression profiles were observed between the mutated neurons and the isogenic control neurons. The RNA profile pertaining to the activities of the nervous system and endoplasmic reticulum homeostasis was identified by gene ontology analysis [83]. The involvement of the ERK and JNK signaling pathways in the neurodegeneration of *SOD1* mutated-motor neurons was unraveled by the RNA sequencing of the *SOD1* E100G mutation carrying motor neurons. This mutation when corrected using CRISPR-Cas9, alleviated the neurite growth and ceased cell death [83].

Recently, *SOD1* A4V or D90A mutation carrying iPSCs showed small aggregates in the cytoplasm and neurites of spinal motor neurons derived from these iPSCs [84]. *SOD1* mutated motor neurons, but not interneurons, depicted neurofilament aggregation and neuronal degeneration, recapitulating cell-type-specific degeneration in ALS. These disease phenotypes were mitigated in isogenic controls using TALEN-mediated homologous recombination. Moreover, the introduction of these mutations to normal PSCs induced similar phenotypes in motor neurons, confirming the cause-effect relationship between the gene mutation and disease phenotypes. Further examination of these neurons revealed that *SOD1* mutations lead to neurofilament aggregation and motor neuron degeneration [84]. Subsequently, another group reported using ALS patient cells carrying heterozygous mutations of *SOD1*^+/A272C^ and *FUS*^+/G1556A^ to generate iPSCs and used the CRISPR-Cas9 system along with single-stranded oligodeoxynucleotide (ssODN) to precisely correct these mutations [82]. These iPSCs were consequently differentiated into motor neurons to carry out genome-wide RNA sequencing analysis. They identified some of the transcripts involved in ALS pathogenesis and provided a platform to study ALS disease mechanisms [82]. A recent study based on whole-genome sequencing identified one demographic variant in the X-linked ATP7A gene, M1311V as a strong candidate in pathogenesis. They corrected this mutation using CRISPR-Cas9 in iPSCs-derived from the patient’s fibroblasts and demonstrated significant restoration of the neuronal defects in these corrected iPSC-derived motor neurons [85]. It has been reported recently that, human motor neurons with CRISPR-Cas9 generated *SOD1*-G93A mutation showed disease-relevant phenotypes including SOD1 aggregation, axonal degeneration, and aberrant synaptic functions [86].

Another major application of patient iPSCs is for drug screening. A study reported the repair of L144FVX mutation in the *SOD1* by insertion of the wild-type *SOD1* along the homologous arms in the iPSCs-derived from the patient [87]. As expected, the corrected iPSCs showed a reversal of the disease phenotype. High throughput screening using these patient iPSCs identified hits for Src/c-Abl pathway as a potential therapeutic target [88]. CRISPR-Cas9 system also showed the capacity of identifying the *C9ORF72* modifiers in the genome-wide gene knockouts. This study pointed out a modifier, TMX2 can modulate the ER-stress signature caused by *C9ORF72* dipeptide-repeat and improve the survival of motor neurons from human *C9ORF72* ALS patient [88]. Motor neurons generated from patient-derived iPSCs carrying *TDP-43* mutation were used to screen chemical compounds leading to the identification of anacardic acid that rescued the abnormal phenotypes [89]. These results indicate that motor neurons derived from patient-specific iPSCs can be used for understanding ALS disease pathogenesis and screening therapeutic agents. In vivo gene editing of *SOD1* mutations using the CRISPR-Cas9 has been studied in ALS mice, which has been shown to extend the lifespan and enhance the functions of the motor neurons [90,91,92].

### 3.2. The Use of Gene Editing and Stem Cell Models for Studying SMA

SMA, a recessive genetic disorder affecting 1 in 6000 children, is characterized by muscle atrophy due to loss of spinal motor neurons [93]. In 1995, *Survival Motor Neuron 1 (SMN1)* gene was first identified as the SMA-determining gene [94]. Unlike rodents that have a single form of the SMN gene, humans have two forms of this gene: *SMN1* and *SMN2.* Though these two genes are similar, there is an important substitution mutation Cytosine (C)- to-Thymine (T) in *SMN2* exon 7 at position 6 which causes disrupted splicing of exon 7 and leads to the formation of an unstable SMN 7 protein [95,96]. About 95% of patients inherit the homozygous deletion of the *SMN1* gene. Though patients still have the *SMN2* gene, *SMN2* cannot compensate for the loss of *SMN1* and patients will have reduced levels of functional SMN protein, leading to the degeneration of spinal motor neurons and subsequent impaired neuromuscular junction and muscle atrophy. Why the deficiency of SMN protein, which is ubiquitously expressed protein in the CNS, results in the specific degeneration of spinal motor neurons remains largely unclear.

Given that humans are unique in having two *SMN* gene isoforms, it is important to build SMA models that reflect the human genotype. Patient-specific iPSCs were established by reprogramming fibroblasts from a 3-year-old boy suffering from SMA type I patient via lentiviral transduction in 2009 [97]. These iPSCs were then differentiated into spinal motor neurons that exhibited selective degeneration. Similarly, SMA iPSCs have been generated from patients with different SMA types [98,99,100,101,102,103]. It has been shown that the SMN levels are much lower in cultures derived from SMA type I iPSCs than those from SMA type III iPSCs [104] as well as the neurite growth deficit was more severe in SMA type I spinal motor neurons, correlating to the disease severity [104]. SMA iPSC-derived motor neurons also exhibit reduced neuromuscular junctions when co-culturing with muscle cells, suggesting impaired connectivity between SMA motor neurons and muscles [105,106]. In addition to iPSCs, the other strategy to generate SMA models is to knock down the *SMN1* gene in hESCs. It was reported that the knockdown of the *SMN1* gene did not interfere with the neural induction and differentiation processes [107]. Notably, it resulted in reduced axonal outgrowth, impaired mitochondrial transport, and increased swellings, recapitulating the motor neuron-specific degeneration. Moreover, these disease-relevant phenotypes are specific to spinal motor neurons and can be rescued when the SMN levels are restored [107].

Since human PSCs can differentiate into different types of cells, SMA PSCs can be differentiated into different types of neurons, as well as glial cells [101,108,109] to dissect specific changes in spinal motor neurons. However, an accurate motor neuron reporter system is critical for live-cell imaging to compare the function and viability between motor neurons and other types of neurons in live cells. To target the spinal motor neurons specifically, the HB9 gene expressed specifically in motor neurons at early stages has been used to construct reporter lines [110]. Another gene named vesicular acetylcholine transferase (VACHT) expressed in all cholinergic neurons has also been selected for generating transgenic lines. These reporter lines are created either by knocking in a fluorescent reporter gene (TdTomato) or Cre/CreERT2 recombinase into the reporter genomic loci [110]. The HB9-tdTomato reporter line is compatible with Fluorescent Activated Cell Sorting (FACS) and capable of live imaging with high magnification, while the fluorescence is weak under low-intensity illumination. The tamoxifen-inducible HB9-CreERT2 reporter cell line has brighter fluorescence although only 30–40% HB9^+^ neurons are effectively labeled, suggesting that this inducible system needed further improvement. Interestingly, the fluorescence intensity of the VACHT-Cre/tdTomato reporter cell line was twice in magnitude compared to the HB9-tdTomato line, which makes it a better choice for live cell imaging [110]. As VACHT is expressed in all cholinergic neurons, combination with other motor neuron markers will be needed to confirm the spinal motor neuron identity.

Considering that SMA is caused by loss of SMN function, the therapeutic strategies for SMA have been focused on increasing the functional SMN protein; including increasing the *SMN2* promotor activity, targeting the splicing, increasing SMN protein stability, and gene therapy to deliver the *SMN* gene [111,112,113]. The use of gene editing in iPSCs for treating SMA is gaining popularity in recent times. A study showed the in situ gene conversion of *SMN2* to *SMN1* using the CRISPR-Cpf1 in SMA iPSCs. These iPSCs possessed the normal karyotype and rescued the SMN expression and gems localization in the motor neurons. A different strategy was reported which involved correcting the *SMN2* disrupted splicing using CRISPR-Cas9 in SMA iPSCs. This group targeted the intronic splicing-regulatory elements, resulting in higher expression of the functional SMN protein [114]. For screening therapeutic agents to target the *SMN2* gene, one important application of gene editing in SMA is to build *SMN2* reporter lines. Using the CRISPR-Cas9 system to specifically target the *SMN2* gene, an *SMN2*-GFP reporter in HEK cells was generated to test drugs [115]. Cysteine protease inhibitors were identified to increase the stability of the functional protein and alleviated neuropathy as well as mitochondriopathy [115]. Future utilization of SMA iPSCs to generate *SMN2* reporter lines, as well as reporters for other targets, will allow researchers to screen drug libraries in *SMN-*deficient human motor neurons to identify potential therapeutic agents for SMA. Overall, these reports provide a proof-of-concept to support the possibility of direct gene editing of motor neuron disease-related genes (e.g., *SMN2* and *SOD1*) for exploring the treatment of these diseases.

### 3.3. Other Motor Neuron Diseases

In addition to ALS and SMA, human stem cell models have been established for other motor neuron diseases including hereditary spastic paraplegias (HSPs) (Figure 2). HSPs are a group of genetic disorders that have more than 80 gene loci linked to them and are denoted as *SPG1-80*. HSPs are characterized by spasticity and weakness of hip and leg muscles that are caused by axonal degeneration of cortical motor neurons. HSP patient-specific iPSC lines were generated for multiple form of HSP including *SPG4* [116,117], *SPG3A* [118], *SPG11* [119,120], *SPG15* and *SPG48* [121]. By knocking in disease-related mutations using gene editing, disease-specific phenotypes were recapitulated, providing additional models to study HSPs [122]. An interesting unanswered question for HSPs is how the mutations of divergent protein lead to similar symptoms and axonal degeneration of cortical projection neurons. Using hPSC-based models, multiple pathological pathways have been identified in the patient-relevant human nerve cells including microtubule defects, mitochondrial and lipid dysfunction [123,124]. Using the gene editing strategy, other mutations can be generated, and importantly mutations in patient iPSCs can also be corrected. This will provide unique paradigms to dissect the role of HSP gene mutations in disease phenotypes in HSP.

The patient-specific stem cell models have been used to study various motor neuron diseases. After understanding the underlying neuropathological players associated with each particular disease, these genes need to be manipulated using artificial methods to retrieve their normal expression as observed in the wild type. To achieve this, the gene therapy field has recently been developed and is rapidly flourishing in biomedical research. Several genes responsible for the occurrence of the particular neurodegenerative disease have been identified, for example, *SOD1* for ALS and *SMN* for SMA. Strategies have been developed to compensate for the under or overexpression of these causative genes using ASOs, siRNA, and viral vectors. Gene therapies using these approaches will be discussed in the next section.

## 4. Gene Therapy for Motor Neuron Diseases

Gene therapy is considered a promising area for treating various human diseases. This has opened multiple avenues focusing on correcting the causative gene(s) in certain diseases. It can be used for gene knock-in/knock-out, gene editing, and gene replacement. While designing the strategies for gene therapy, it is pivotal to take into consideration the typical characteristics of the genetic material used, the vehicle used for delivery, and the route of administration. The chemical composition of the nucleic acids like the oligonucleotides or silencing RNA can be tweaked to improve their stability [125]. Among the non-viral-based methods, antisense oligonucleotides (ASOs) are an emerging approach for motor neuron diseases.

The ASOs are 10–25 nucleotides long single-stranded nucleic acids that are designed to attack the mRNA and alter its splicing pattern or cause its degradation by activating the RNase H [126]. The ASOs cannot cross the blood-brain barrier and are delivered intrathecally to reach the brain and the spinal cord through the cerebrospinal fluid to treat neurodegenerative diseases [127]. To treat the SOD1-linked ALS, Biogen Inc. sponsored the phase I/II clinical trials for the SOD1 ASO called Tofersen (NCT02623699). This ASO showed a decline in the SOD1 expression profile in treated patients and was promoted to Phase III clinical trial. Another gene linked to ALS, C9ORF72 was also targeted by ASO and tested in iPSCs, C9ORF72 mutation bearing patient-derived fibroblasts, and mouse models [128]. This C9ORF72 ASO blocked the sequestration of the RNA binding proteins by the C4C2 repeats or by activating the RNase H to cause mRNA degradation [129]. In C9-450 mouse models, this ASO showed mitigation of the cognitive functions due to the expansion of C9ORF72 repeats and also plummeting RNA foci [128]. After testing pre-clinically in BAC transgenic mice, the phase I/II clinical trials (NCT03626012) were sponsored by Ionis Inc. and Biogen Inc. to administer repeated intrathecal injections of IONIS-C9 (BIIB078) to the patients. For treating the SMA, the first FDA-approved ASO gene therapy called nusinersen was developed. Nusinersen is an 18 mer phosphorothioate 2′-O-methoxyethyl (2′-MOE) which binds ISS-N1 present in intron 7 in the SMN gene [130]. This binding promotes the inclusion of exon 7 and thus correcting the defective splicing of the SMN2 pre-mRNA, resulting in increased production of functional SMN protein [131]. After successful pre-clinical trials and Phase II/III clinical trials in 2016, Biogen Inc. and Ionis Inc. commercially marketed nusinersen as SPINRAZA [132].

Though ASOs have many benefits, they require intrathecal administrations which put forward complications in human patients regarding this invasive route of delivery. Also, the ASOs may be confined to the spinal cord and may not reach the brain niche to exert their effects. In contrast to ASOs, the viral delivery system shows widespread delivery in the CNS and can cross the blood-brain barrier. Particularly, the AAV viral vector-mediated delivery shows the high efficiency of transduction, especially in neuronal cells. There are 13 serotypes of AAVs with AAV9 and AAVrh10 exhibiting tropism in the nervous system, especially motor neurons [133,134]. The AAV system has shown positive results in the mouse models of ALS. There was a 39% rise in the median lifespan of SOD1-G93A mice when transfected with AAV9 carrying shRNA silencing the SOD1 [135]. This shRNA-based strategy is under the pre-clinical trials by AveXis Inc. [136]. The AAVrh10 has also been tested extensively in ALS mice models and higher primate models, which showed an increase in mice survival [137,138] and effectiveness in silencing SOD1 [139]. These studies provided a strong foundation for the pre-clinical trial of this approach which is conducted by Voyager Therapeutics Inc. and Apic Bio Inc. Another remarkable application of this AAV system has been designed by combining it with ASOs. The AAV-U7-AS system comprising the AS responsible for the degradation of SOD1 RNA was delivered by U7 small nuclear RNA through injection with AAVrh10 vector in SOD1-G93A mice at two different time points. Injection at birth increased survival by 92% and in 50 days old mice 58% increase in survival was reported [140]. In primary neurons isolated from C9BAC transgenic mice with C9ORF72 mutation, a study silenced the C9ORF72 transgene by rAAV9-mediated delivery of miR sequences in the primary cortical neurons [141]. The results depicted the impairment of the C9ORF72 transgene expression and reduction in poly(GP) dipeptides, supporting the potential value for using rAAVs to treat ALS.

This rAAV-based gene therapy has also been utilized for treating SMA. It has been shown in SMA mice models that the injection of the scAAV SMN through the facial vein led to a rise in the SMN levels in the CNS. Surprisingly, the defect in the neuromuscular connections was also reversed and the life span was increased [142]. Next, the transduction efficiency of the AAV9 was checked in primates and showed the transduction of the motor neurons, glial cells, other regions of the CNS, and skeletal muscles [143]. To test in different species, 5 days-old pigs were also injected with AAV9-GFP [143]. The results obtained confirmed the CNS transduction efficiency of AAV9 and set the stage for its delivery in humans. In 2014, the first phase I clinical trials were undertaken by AveXis Inc. using a single intravenous injection of scAAV9-SMN in 15 SMA type I patients [144]. The majority of the patients in the trial accomplished the major motor milestones and terminated the use of artificial respiratory support. This was a remarkable feat for the AAV9 gene therapy for treating the SMA. There were no side effects observed in these patients, except two of them had elevated serum aminotransferase levels, which was consequently regulated by prednisolone drug. In 2019, the USFDA approved ZOLGENSMA by AveXis Inc. as scAAV9 therapy to treat SMA.

## 5. Conclusions and Future Aspects

In this review, we discussed the stem cell models for motor neuron diseases and different gene editing tools developed over the years. We also discussed the applications of gene editing and gene therapy, especially the combination of these approaches and stem cell modeling for motor neuron diseases. Pluripotent stem cells, especially patient-specific iPSCs offer unlimited sources to generate patient-derived neurons for studying neurological diseases including motor neuron diseases. One major challenge in stem cell models is to efficiently differentiate and purify the target neuronal subtypes. Recent studies have greatly improved the efficacy in generating spinal motor neurons [98,145], though the highly efficient generation of cortical motor neurons awaits further investigation. Another challenge is to recapitulate synaptic defects and to mimic the circuitry in stem cell models. These could be addressed by generating three-dimensional models, or by transplanting stem cell-derived neural cells to animals to test their maturation, connections, and defects in vivo environment.

With the development of gene editing tools like ZFNs, TALENs, and CRISPR-Cas9, there was a significant improvement in the gene editing efficiency, allowing the generation of isogenic lines and reporter lines, which facilitate the study of motor neuron diseases. Although CRISPR-Cas9 is the gene editing tool preferred by researchers for a great number of applications there are a few concerns associated with it. One of the major concerns is the off-target effect leading to undesired mutagenesis. Another shortcoming that has been pointed out is the low frequency of homologous recombination. To enhance the homologous recombination-mediated repair activity followed by DNA nicking, molecules that suppress the NHEJ pathway could be utilized [146]; also, the cell cycle can be synchronized when delivering this system in the cell [147]. As researchers continue to edit the human PSC genome, it will be easier to assess mutations in various cell lines with multifarious genetic backgrounds. Another approach is to correct the disease mutation by gene editing in the patient-specific iPSCs and to knock in a disease-causing mutation into the normal cells, which will confirm the role of the specific mutation in a disease. The in vivo gene editing is yet another luring field for many researchers, but there are ethical and technical difficulties associated with it [148,149]. All in all, the gene editing applications would have noteworthy contributions in reversing the disease phenotypes as well as in discovering novel therapeutic targets, leading to effective treatment possibilities.

The area of gene therapy is promising for treating neurodegenerative diseases especially ALS and SMA. The ASOs and the siRNAs have been tested and used in clinical trials for ALS and SMA. The viral-based methods have been proven advantageous for their stable expression in the nervous system. The AAV-mediated delivery is the latest and advanced option for gene therapy, not only in animal models but also in clinical trials. AAVs can target specific cell types and carry out the delivery of genes in a precise manner. Furthermore, the editing of an endogenous gene provides a new strategy for gene therapy, though more testing on its safety and efficacy in vivo is required. Further investigation in this area, as well as the combination of gene editing and stem cell technology, can advance our understanding and provide valuable insights into developing therapies for motor neuron diseases.

## Data Availability

Not applicable.
